# A retrospective study of SARS-CoV-2 seroprevalence in dogs and cats in the Community of Madrid, Spain

**DOI:** 10.3389/fmicb.2023.1264172

**Published:** 2023-10-05

**Authors:** Lidia Sánchez-Morales, José M. Sánchez-Vizcaíno, Lucas Domínguez, Sandra Barroso-Arévalo

**Affiliations:** ^1^VISAVET Health Surveillance Centre, Complutense University of Madrid, Madrid, Spain; ^2^Department of Animal Health, Faculty of Veterinary Science, Complutense University of Madrid, Madrid, Spain

**Keywords:** seroprevalence, SARS-CoV-2, domestic animals, antibodies, Spain

## Abstract

To date, susceptibility to SARS-CoV-2 infection in domestic animals including cats and dogs has been described. However, it is important to carry out passive surveillance of these animals to be aware of any changes in the outcomes of the disease in these species that may occur. In this study, we have performed a retrospective study in which we analyzed sera (*n* = 1,640) from random animals: dogs (*n* = 1,381) and cats (*n* = 259) belonging to both homes (*n* = 1,533) and animal protection centers (*n* = 107) in the Community of Madrid, Spain. Neutralizing antibodies were evaluated between November 2021 and May 2022 using a surrogate ELISA kit to determine the seroprevalence. Based on the results obtained, a few animals (both cats and dogs) presented neutralizing antibodies to SARS-CoV-2 (2.3%), all of them from private owners. However, the seroprevalence in cats (4.6%) resulted to be almost twice as much as in dogs (1.9%) which reinforces that cats’ susceptibility to the infection seems higher than in the case of dogs, maybe due to the lower ACE2 expression of the dogs in the respiratory tract. These findings also confirm that the probability of infection is considerably higher in domestic animals in close contact with infected owners, compared to animals living in animal shelters whose contact with humans is markedly lower.

## 1. Introduction

Since the beginning of the pandemic (December 2019) caused by the severe acute respiratory syndrome coronavirus 2 (SARS-CoV-2), more than 6 million deaths and over 664 million cases have been reported worldwide (World Health Organization [WHO], 2022). Many vaccine prototypes have been developed for the coronavirus disease 2019 (COVID-19) in a very short time (Hahn and Wiley, 2022) and more than 13,000 million vaccine doses have already been administered (World Health Organization [WHO], 2022). COVID-19 is a disease of potential zoonotic origin whose host affinity is determined by the virus’ spike protein (S) (Wan et al., 2020). This protein binds to host cells through the angiotensin-converting enzyme 2 (ACE2) protein receptor which is present in many animal species (Wan et al., 2020). The variety of animal species in which the natural infection with SARS-CoV-2 virus has been detected ranges from domestic to wild animals (Sit et al., 2020; Palmer et al., 2021). In addition, further species have been reported to be susceptible to the virus according to experimental studies such as domestic swine (Pickering et al., 2021) and cattle or goats (Bosco-Lauth et al., 2021). The natural detection of the disease in animals as well as their potential role as intermediate or reservoir hosts led to the need of studying the disease in different experimental animal models (Halfmann et al., 2020; Shi et al., 2020).

Although domestic animals do not seem to suffer from serious consequences in terms of clinical signs from SARS-CoV-2 infection, it is important to carry out active and passive surveillance programs to monitor the presence of the disease as well as the infection trends in the different susceptible animal species. In addition to domestic animals, the virus has been detected in wild species such as white-tailed deer (*Odocoileus virginianus*) (Palmer et al., 2021) and mink (Neovison vison) on mink farms (Larsen et al., 2021; Oude Munnink et al., 2021). In these farms, it was found that the disease had passed from humans to mink and back to humans. Another novel finding is the study of Sila et al. (2022) that hypothesizes the transmission of the disease from an infected cat with SARS-CoV-2 to its veterinarian.

The presence of an active infection in these animals can be evaluated by the detection of viral RNA by a reverse transcription quantitative real-time polymerase chain reaction (RT-qPCR), commonly from samples such as nasopharyngeal or oropharyngeal swabs (Sule and Oluwayelu, 2020). However, viral RNA can only be determined a few days after the infection (Meekins et al., 2021), whereas antibody detection can be performed a long time after the infective period, in order to elucidate whether animals have been exposed to the virus or not. The evidence of infection has already been confirmed by the detection of anti-SARS-CoV-2 antibodies in several field studies conducted on domestic animals in continuous contact with their RT-qPCR-positive owners (Michelitsch et al., 2020, 2021; Patterson et al., 2020; Zhang et al., 2020; Dileepan et al., 2021; Fritz et al., 2021; Stevanovic et al., 2021).

Given all these events related to animals, as well as the continuous appearance of new variants, the importance of sanitary surveillance of the disease in animals is emphasized. To improve the current knowledge on this topic, the present study retrospectively evaluates the seroprevalence of the infection in dogs and cats in the Community of Madrid, a region with a high incidence of disease in humans.

## 2. Materials and methods

A total of 1,640 serum samples randomly chosen from companion animals (1,381 dogs and 259 cats) were collected from November 2021 to May 2022 in the Community of Madrid and sent to the VISAVET Health Surveillance Center for their analysis. The samples were transported from the laboratory to VISAVET center by a transport company under the regulations stated in UN3373. Upon arrival at the center, samples were taken to the biosafety level 3 (BLS3) facilities and stored at 4°C for processing and further analysis. The information available for each of the samples was: the animal species, the date of sampling and their origin [households or animal protection centers (APCs)] with no data regarding clinical signs or contact with positive owners/caretakers.

A detection of antibodies by SARS-CoV-2 Neutralizing Antibody Detection Kit (GenScript Inc., Piscataway, NJ, United States) was made. The procedure was carried out on all the samples using the GenScript cPass™ SARS-CoV-2 Neutralizing Antibody Detection Kit in which the protein-protein interaction between HRP-RBD (horseradish peroxidase-receptor binding domain) and human angiotensin-converting enzyme II (hACE2) can be blocked by neutralizing antibodies against SARS-CoV-2 RBD, which was already validated (Perera et al., 2021). Samples and controls are diluted with a sample dilution buffer and pre-incubated with the HRP-RBD diluted solution to allow the binding of the circulating neutralization antibodies to HRP-RBD. The mixture is then added to the capture plate which is pre-coated with the hACE2 protein and incubated at. The unbound HRP-RBD as well as any HRP-RBD bound to non-neutralizing antibody will be captured on the plate, while the circulating neutralizing antibodies HRP-RBD complexes remain in the supernatant and get removed during washing. Following a four-wash cycle, solution is added and incubated in dark and then followed by the Stop Solution. The reaction is quenched, and the color turns yellow. The absorbance of the sample is inversely dependents on the titer of the anti-SARS-CoV-2 neutralizing antibodies. Results of each individual samples was calculated using the following formula:


$$\mathrm{Inhibition}\left(\%\right)=\left(1-\left(\frac{\mathrm{OD}\,\mathrm{value}\,\mathrm{of}\,\mathrm{sample}}{\mathrm{OD}\,\mathrm{value}\,\mathrm{of}\,\mathrm{Negative}\,\mathrm{control}}\right)\times 100\right)$$

Samples presenting a cutoff higher or equal to 30% are considered positive results indicating the presence of SARS-CoV-2 neutralizing antibodies and lower than 30% are considered negative results according to the manufacturer’s instructions. This negative result indicates the absence or a level of SARS-CoV-2 neutralizing antibodies lower than the limit of detection, but it can also appear in samples taken during an acute infection before antibody seroconversion.

For the statistical analysis, a Chi-square test was performed to assess if there was any difference in the proportion of seropositivity to SARS-CoV-2 between species. All statistical analyses were performed with IBM^®^ SPSS^®^ Statistics v. 23.0.

## 3. Results

Thirty-eight samples from animals living in houses (26 dogs and 12 cats) out of the total analyzed (*n* = 1,640) were positive (inhibition percentages between 30 and 94%) for the SARS-CoV-2 Neutralizing Antibody Detection kit, which represented a seroprevalence of 0.023 (38/1,640) of the total (CI 95%: 0.017–0.032). The results depending on species and their origin are represented in Table 1.

**TABLE 1:** Positive animals to the presence of neutralizing antibodies out of the total depending on origin.

Species	Seropositive animals/total	Origin	No. of sera	No. of positive samples (%) based on species and origin
Dogs	26/1,381 (1.9%)	APCs	80	0 (0.0%)
		Households	1,301	26 (2%)
Cats	12/259 (4.6%)	APCs	27	0 (0.0%)
		Households	232	12 (5.2%)

**FIGURE 1:**
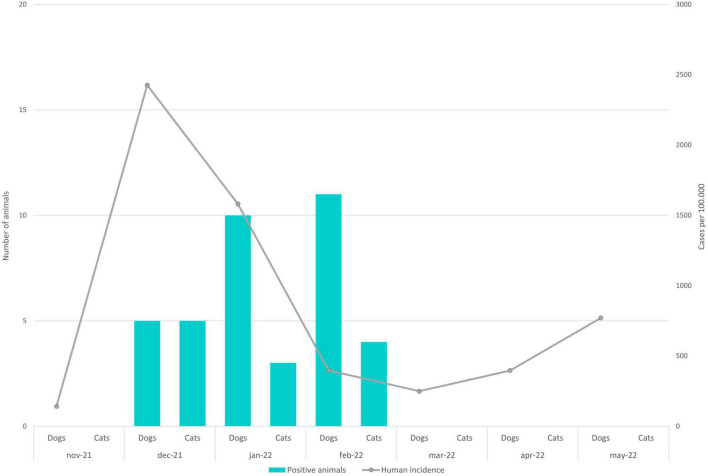
Graphical representation showing on a monthly basis the number of dogs and cats presenting neutralizing antibodies against SARS-CoV-2. In addition, the accumulated incidence in humans during the same months in the Community of Madrid is represented.

The number of positive animals sampled over the months as well as accumulative incidence in humans along those months are shown in Figure 1.

A significant difference between seropositivity and species (dogs and cats) was found (*p*-value = 0.007). The seroprevalence in cats reached 0.046 (CI 95%: 0.027–0.079) while in dogs it was 0.019 (CI 95%: 0.013–0.027). All the positive samples, both from cats and dogs proceeded from private owners living in houses.

## 4. Discussion

Numerous studies have already demonstrated the susceptibility of dogs and cats to SARS-CoV-2 infection (Dileepan et al., 2021; Fritz et al., 2021), so the control and surveillance of the disease in these companion animals is considered of a great importance. In this study, we have evidenced a low seroprevalence in cats and dogs, being all the positive samples from household animals. Sampling of the animals was carried out mainly between November 2021 and February 2022, dates which coincide with the time when the Omicron variant appeared in Spain. Moreover, in mid-December 2021, almost 50% of the sequenced samples in humans belonged to the Omicron variant, while the rest of the sequenced samples belonged to the Delta variant. One month later, the number of cases per 100,000 inhabitants increased exponentially and by mid-January 2022, 95% of the sequenced samples were of the Omicron variant (World Health Organization [WHO], 2022). In the present retrospective randomized study, we have no information on the health status of the animal owners or the degree of contact with the animals. Even though we do not have this information, the appearance of the Omicron variant greatly increased the incidence in people, so this, together with the dates, suggests that in this sampling we could be detecting antibodies to this new variant of concern. However, the Omicron variant was already found to have a lower immunogenicity than previous variants and does not seem to induce a strong antibody response, (Sánchez-Morales et al., 2022; Tyson et al., 2023) which could mean that the antibodies we are detecting are derived from previous variants. However, the emergence of the Omicron variant greatly increased the incidence in people, so this, together with sampling dates, suggests that we may be detecting antibodies to this variant of concern.

The fact that only household animals tested positive for this study is consistent with numerous studies in which a continued exposure of animals to infected people seems to be a determining factor (Dileepan et al., 2021; Bessière et al., 2022). None of the animals from APCs showed neutralizing antibodies, which might be due to the fact that these animals do not have as continuous and close contact with people as household animals do with their owners. In animal shelters, animals have much more limited contact with their caretakers at specific times. The same negative results were obtained in a study carried out in Italy in stray cats (Stranieri et al., 2022) and similar, with a very low seroprevalence (0.009%) (van der Leij et al., 2021) in animals from APCs in Netherlands.

In addition, we have observed that the prevalence of the presence of neutralizing antibodies in cats (4.6%) was higher than in dogs (1.9%). These results coincide with seroprevalence studies carried out in Minnesota (Dileepan et al., 2021), Italy (Patterson et al., 2020), the UK (Smith et al., 2021), and France (Bessière et al., 2022) with much higher seroprevalence percentages in cats than dogs. In fact, in experimental infection studies, it has been demonstrated that cats are much more susceptible to the infection than dogs (Shi et al., 2020), they can spread SARS-CoV-2 to other cats and sometimes even develop lesions (Bao et al., 2021; Chiba et al., 2021) and have symptoms (Natale et al., 2021). All these results could be related to the lower expression of ACE2 receptors in the respiratory tract of dogs (Zhai et al., 2020). In addition, the ACE2 of dogs, compared to humans, has five mutations while cats only have four (Zhai et al., 2020), which could explain the higher susceptibility to the infection in this species.

The seroprevalence results observed in domestic cats in this study in Madrid (4.6%), were similar to those obtained in Italy in 2020 (5.8%) (Patterson et al., 2020). On the other hand, there were countries in which seroprevalence results were much higher than ours, being the majority of them of domestic cats such as in Minnesota (11–12%) (Dileepan et al., 2021) or France (8.4%) (Bessière et al., 2022). The higher seroprevalence in France may be related to the higher number of SARS-CoV-2 human cases than in Spain, being the cumulative incidence in France in January 2022 of 7.200 cases per 100.000 habitants (World Health Organization [WHO], 2022). There were also studies in which the percentage of seroprevalence was lower than in our study such as Portugal (1.7%) (Oliveira et al., 2022) or Poland (1.79%) (Pomorska-Mól et al., 2021), coinciding with countries in which the number of SARS-CoV-2 cases is lower than in Spain (World Health Organization [WHO], 2022).

The results obtained in this study from a random sampling of dogs and cats in the Autonomous Community of Madrid indicate that the virus is circulating among domestic animals, being cats more susceptible or exposed than dogs and that they are able to develop neutralizing antibodies. These results, together with all the studies that have been carried out to date, both natural and experimental infection in animals, lead us to emphasize the importance of active and passive surveillance of this disease in both wild and domestic animals. In this way, we will be able to learn the behavior of this virus in each of the animal species, as well as the possible changes that may arise.

## Data availability statement

The original contributions presented in this study are included in the article/supplementary material, further inquiries can be directed to the corresponding author.

## Ethics statement

The animal studies were approved by the Complutense University of Madrid’s Ethics Committee for Animal Experiments (Project License 14/2020). The studies were conducted in accordance with the local legislation and institutional requirements. Written informed consent was obtained from the owners for the participation of their animals in this study.

## Author contributions

LS-M: Methodology, Writing – original draft. JS-V: Conceptualization, Funding acquisition, Writing – review and editing. LD: Funding acquisition, Writing – review and editing. SB-A: Conceptualization, Writing – review and editing.
